# A Simple Synthesis of Reduction-Responsive Acrylamide-Type Nanogels for miRNA Delivery

**DOI:** 10.3390/molecules28020761

**Published:** 2023-01-12

**Authors:** Ali Maruf, Małgorzata Milewska, Anna Lalik, Sebastian Student, Ilona Wandzik

**Affiliations:** 1 Department of Organic Chemistry, Bioorganic Chemistry and Biotechnology, Faculty of Chemistry, Silesian University of Technology, Krzywoustego 4, 44-100 Gliwice, Poland; 2 Biotechnology Center, Silesian University of Technology, Krzywoustego 8, 44-100 Gliwice, Poland; 3 Department of Systems Biology and Engineering, Faculty of Automatic Control, Electronics and Computer Science, Silesian University of Technology, Akademicka 16, 44-100 Gliwice, Poland

**Keywords:** drug delivery, glutathione, miRNA, nanogel

## Abstract

MicroRNAs (miRNAs) have great therapeutic potential; however, their delivery still faces huge challenges, especially given the short half-life of naked miRNAs due to rapid hydrolysis or inactivation by abundant nucleases in the systemic circulation. Therefore, the search for reliable miRNA delivery systems is crucial. Nanogels are one of the more effective nanocarriers because they are biocompatible and have a high drug-loading capacity. In this study, acrylamide-based nanogels containing cationic groups and redox-sensitive crosslinkers were developed for cellular delivery of anti-miR21 (a-miR21). To achieve this, post-polymerization loading of a-miR21 oligonucleotides into nanogels was performed by utilizing the electrostatic interaction between positively charged nanogels and negatively charged oligonucleotides. Different molar ratios of the amine groups (N) on the cationic nanogel and phosphate groups (P) on the miRNA were investigated. An N/P ratio of 2 allowed high miRNA loading capacity (MLC, 6.7% *w*/*w*) and miRNA loading efficiency (MLE, 99.7% *w*/*w*). Successful miRNA loading was confirmed by dynamic light scattering (DLS) and electrophoretic light scattering (ELS) measurements. miRNA-loaded nanogels (NG/miRNA) formed stable dispersions in biological media and showed an enhanced miRNA release profile in the presence of glutathione (GSH). Moreover, the addition of heparin to dissociate the miRNA from the cationic nanogels resulted in the complete release of miRNA. Lastly, a cell uptake study indicated that NG/miRNA could be easily taken up by cancer cells.

## 1. Introduction

MicroRNAs (miRNAs) are naturally occurring noncoding RNAs with short 18–25 nucleotide sequences that regulate gene expression at the post-transcriptional level [1]. Interest in developing miRNA-based therapies for cancer has risen sharply as growing evidence shows that miRNAs play important roles in regulating various cellular mechanisms (e.g., proliferation, programmed cell death, carcinogenesis, and tumor metastasis) [2,3,4]. However, naked extracellular miRNAs have a short half-life due to degradation by extracellular nucleases that are abundantly present in the blood plasma. Depending on the types of miRNAs, their half-lives range from 1.5 h to 13 h at 37 °C [5]. Moreover, miRNAs are not readily taken up by cells due to their hydrophilic nature, negative surface charge, and relatively high molecular weight (~7 kDa), which results in poor cellular uptake and internalization [6]. To address these problems various delivery systems have been developed during the last 20 years. Typically, there are two categories of miRNA delivery systems: viral and nonviral, with the most popular approach being viral vectors [7,8,9]. However, conventional viral vector systems are costly and difficult to produce on a large scale. Therefore, nonviral approaches are being extensively explored, since they offer advantages including easily tunable physicochemical properties, simplified production, high loading capacity, and reduced biosafety concerns. Major approaches in this category involve the combination of nucleic acids with transfection reagents, such as inorganic, lipid, and polymeric-based nanoparticles [7,8,9]. Among polymeric nanocarriers, the most exciting innovations in nanomedicine for gene delivery are nanogels, because they are biocompatible, possess high loading capacity, and have potential responsivity to environmental stimuli [10,11]. Recent studies have shown the promising use of synthetic nanogels as miRNA [12,13,14,15,16,17], siRNA [18], mRNA [19], and pDNA [20] delivery systems.

One of the first examples of an miRNA-based nanogel system was designed by Liu et al. [16]; a-miR21 was encapsulated within a polymer network by in situ polymerization of acrylamide and cationic N-(3-aminopropyl)methacrylamide, with ethylene glycol dimethacrylate as a crosslinker. According to the authors’ hypothesis, nanocapsules containing miRNA were stable in physiological conditions (pH 7.4) and degraded at the lowered pH present in endosomes, releasing miRNA into the cytoplasm.

As commonly performed for other therapeutic genes, loading of miRNA is usually based on the controlled self-assembly of nanogel cationic groups and the anionic phosphate groups of nucleic acids by electrostatic complexation. The exception is the study presented by Dispenza et al. [17], who covalently conjugated a-miR31 into a poly(N-vinyl pyrrolidone)-based nanogel. The authors demonstrated that the covalent linkage of the miRNA to these nanogels does not affect its functionality in vitro. 

Several examples of miRNA delivery systems are based on nanogels crosslinked by disulfide bonds, thus forming a redox-sensitive system [12,13,14,15]. Among the various stimuli, the reductive environment caused by the presence of glutathione (GSH) is the most prominent and characteristically distinctive stimulus in cancer cells. The cleavage of disulfide bonds in the intracellular compartments is favored because of high concentrations of GSH within cells (2–10 mM) in comparison to micromolar levels (2–20 µM) in the blood plasma [21,22].

Shatsberg et al. developed reduction-responsive degradable nanogels, which were based on polyglycerol scaffold-containing amino groups [12]. Nanogels were prepared using an inverse nanoprecipitation technique, and miR34a was loaded by electrostatic interaction. The results showed that the nanogel with an optimal content of miR34a could inhibit glioblastoma progression by ~70% within 20 days post treatment. Javanmardi et al. developed PEGylated polyethylenimine redox-sensitive nanogels to encapsulate a-miR21 for the treatment of ovarian cancer in vitro [13,15]. Tumor cell apoptosis and suppression of tumor-associated angiogenesis were conducted on cis-Pt-sensitive ovarian cancer cells and were compared with the resistant cells, for which cisplatin cytotoxicity was remarkably enhanced after a-miR21-loaded nanogel treatment.

In another study, redox-sensitive nanogels were applied to deliver miR34a into transfection-resistant multiple myeloma cells [14]. Thiolated poly(glycidol)-based nanogels with covalently bound positively charged cell-penetrating peptides served as complexation agents for miRNA. Such complexation increased the amount of delivered miRNA, the degree of target gene regulation, and the inhibition of cell viability.

**Figure 1:**
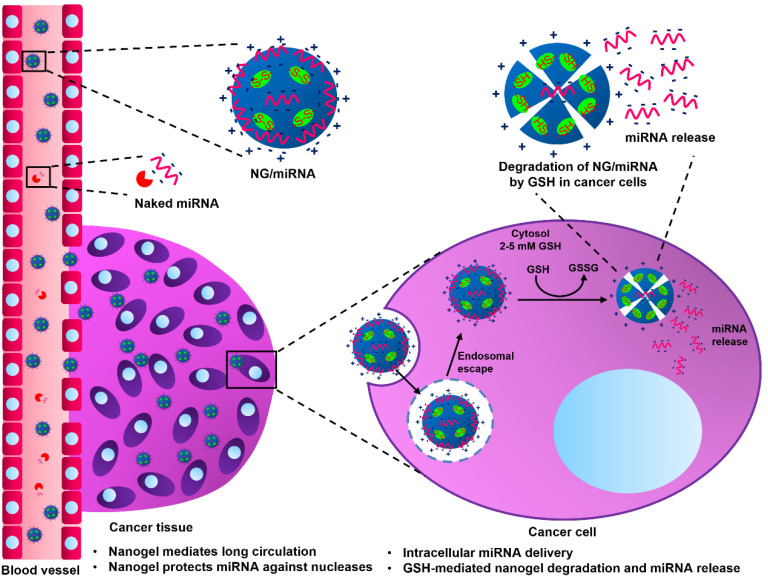
A schematic diagram of NG/miRNA in systemic circulation, miRNA protection against nucleases, enhanced permeability and retention (EPR)-mediated nanogel delivery to cancer tissue, cellular uptake, and intracellular miRNA release induced by GSH in cancer cells.

While the effectiveness of different miRNAs and their role as either oncogenes or tumor suppressors under certain conditions has been well established, many studies on synthetic smart nanogels have neglected to explicate the connection between miRNA loading capacity and release profile with different N/P ratios, either with or without the presence of biological stimuli [12,13,14,15,16,17,18,19,20], which is the main focus of this study. In the current study, we developed biodegradable reduction-sensitive nanogels for miRNA encapsulating and enhancing miRNA release in a cancer-mimicking environment where the GSH level is elevated (Figure 1). The study on miRNA release was also carried out in a GSH-free environment for comparison. Nanogels were synthesized via photoinitiated free-radical polymerization (FRP) of *N,N*-dimethylacrylamide (DMAM) and [2-(acryloyloxy)ethyl]trimethylammonium chloride (ATC) in the presence of a disulfide-type crosslinker. The cationic monomer, ATC, was chosen to provide the electrostatic interactions with the negatively charged miRNA. Anti-miR21 (a-miR21) oligonucleotide was used as a model of miRNA-based therapies due to the fact that miR21 is known as an oncogenic miRNA, which is overexpressed in almost all cancers, and silencing miR21 can affect viability, apoptosis, and the cell cycle [23].

## 2. Results and Discussions

### 2.1. Synthesis and Characterization of NG/miRNA

Stimulus-responsive nanogels have been reported to be effective drug/gene delivery systems for the diagnosis and treatment of various human diseases [10,11]. In our previous study, we developed acrylamide-based trehalose-containing nanogels for autophagy modulation [24] and anionic biodegradable nanogels with high doxorubicin (DOX) loading capacity to prevent DOX from aggregating in biological media and enhance the release of DOX via pH/reduction stimuli [25]. In the design of miRNA-loaded nanogels, we used the same synthetic approach, the only differences being the monomer composition and initiator concentration. In this context, we synthesized reduction-responsive nanogels containing DMAM as the main monomer, [2-(acryloyloxy)ethyl]trimethylammonium chloride (ATC) as the cationic monomer, and *N*,*N*′-bis(acryloyl)cystamine (CBA) as a degradable crosslinker. Nanogels were synthesized via FRP in inverse water-in-oil (w/o) mini-emulsions under LED irradiation at room temperature for 30 min in yield of 56%. Details on monomer feed compositions are shown in Table S1.

The a-miR21 oligonucleotide was loaded into the nanogel in the post-polymerization process (Table S2). Three different compositions were prepared at different N/P molar ratios: 2, 5, and 10 (Table 1). The N/P ratio is defined as the molar ratio between the amine groups (N) on the cationic nanogel and the phosphate groups (P) on the miRNA. 

**Figure 2:**
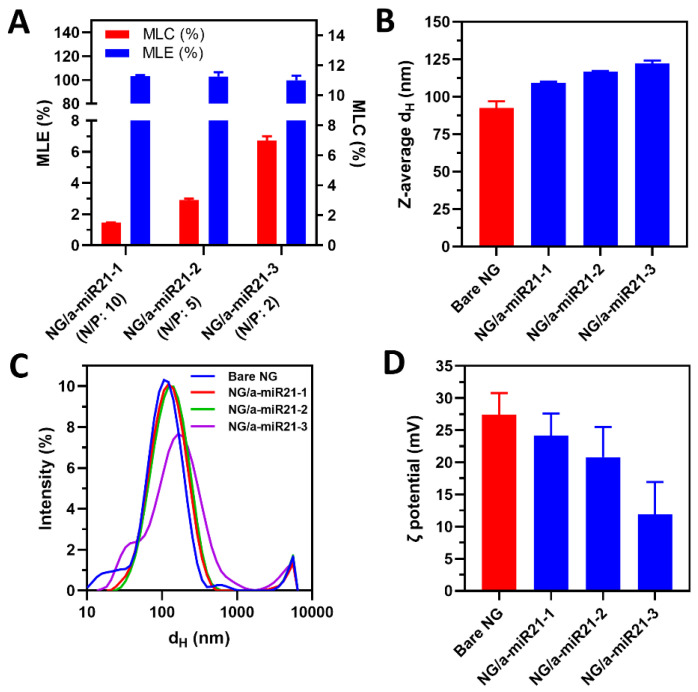
NG/miRNA characterization. (**A**) MLC and MLE of NG/a-miR21-1,2,3. (**B**,**C**) Z-average hydrodynamic diameter (d_H_) of bare NG and NG/a-miR21-1,2,3 in 1 mM KCl measured by DLS. (**D**) ζ potential of bare NG and NG/a-miR21-1,2,3 nanogels in 1 mM KCl measured by ELS. Data are reported as the mean ± SD (n = 4).

The loading of a-miR21 at various N/P ratios was investigated by measurement of fluorescence intensity of nanogels loaded with Cy5-a-miR21 using a standard curve (Figures S1 and S2). In this regard, a relatively high miRNA loading capacity (MLC) of 6.7% *w/w* was achieved for NG/a-miR21-3. On the other hand, NG/a-miR21-1 and NG/a-miR21-2 indicated much lower MLCs of 1.4 and 2.8% *w*/*w*, respectively (Table 1, Figure 2A).

Interestingly, miRNA loading efficiency (MLE) was still guaranteed to be ~100% with the N/P ratio reduced from 10 to 2, which indicated that the negatively charged miRNAs possessed a strong electrostatic interaction with the positively charged nanogels for NG/a-miR21-1,2,3 (Table 1, Figure 2A).

**Figure 3:**
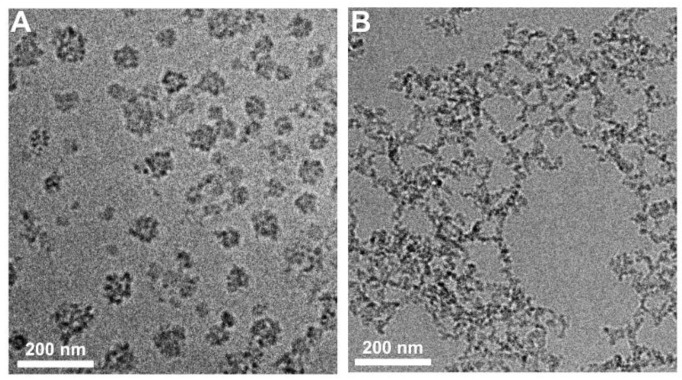
Cryo-TEM micrographs of NG/a-miR21-3 without (**A**) and with (**B**) GSH treatment. Scale bar = 200 nm.

Colloidal properties, such as the particle size and ζ potential of the nanogels, are among the factors that determine stability, transfection efficiency, and biocompatibility. Particle size and ζ potential values were determined before and after loading of a-miR21 at N/P = 2, 5, and 10. Bare NG had an average hydrodynamic diameter (d_H_) of ~90 nm. Increasing the N/P molar ratio from 2 to 10 resulted in a decrease in nanogel size from 122 to 109 nm (Table 1, Figure 2B,C). The ζ potential of bare NG (~27 mV) decreased to 24, 21, and 12 mV after successful miRNA loading into NG/a-miR21-1, NG/a-miR21-2, and NG/a-miR21-3, respectively (Table 1, Figure 2D), due to the neutralizing effects from the negatively charged miRNA. As confirmed by cryogenic transmission electron microscopy (cryo-TEM), NG/a-miR21-3 had a spherical shape with a diameter of about 100 nm (Figure 3A). GSH treatment of NG/a-miR21-3 led to the disintegration of the nanogel network, indicating the cleavage of disulfide crosslinks (Figure 3B).

The optimal size of nanoparticles to acquire the EPR effect in solid tumors is estimated in the range of 100 to 200 nm, bypassing the filtration barriers of the spleen and liver [26]. Additionally, slightly negative or neutral surface charges of nanoparticles are essential to achieve high plasma half-lives [26]. Therefore, NG/a-miR21-3 with an average d_H_ of 122 nm and ζ potential of 11.9 mV is the best candidate to achieve both EPR-mediated tumor penetration and an increase in half-lives.

**Figure 4:**
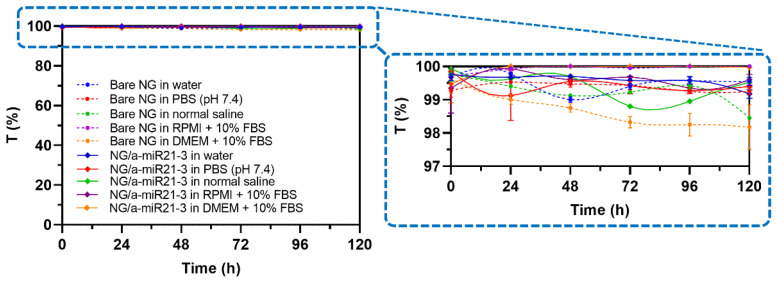
Transmittance values of bare NG and NG/a-miR21-3 dispersions for assessing their colloidal stability in different biological media at a working concentration of 100 µg/mL for 5 days at 37 °C. Data are reported as the mean ± SD (n = 4).

In order to deliver miRNA effectively, nanocarrier systems must be colloidally stable in biological media for both in vitro and in vivo study purposes. According to our previous study, unstable colloidal systems can be easily observed via their transmittance (T) value change in time, in which a T value above ~70% is regarded as stable dispersion. T values below 70% typically show visible aggregates of colloidal systems that are prone to sedimentation. According to the stability study, both bare NG and NG/a-miR21-3 at a concentration of 100 µg/mL had excellent colloidal stability in five different media at 37 °C for 5 days as indicated by their T values (>98%) (Figure 4). Of the media selected, two contained fetal bovine serum (FBS).

### 2.2. miRNA Release Study

The miRNA release profile from nanogel systems is usually not fully explained or not addressed. In this study, we investigated the release rate of miRNA from degradable nanogels in normal and GSH-containing cancer-mimicking environments.

**Figure 5:**
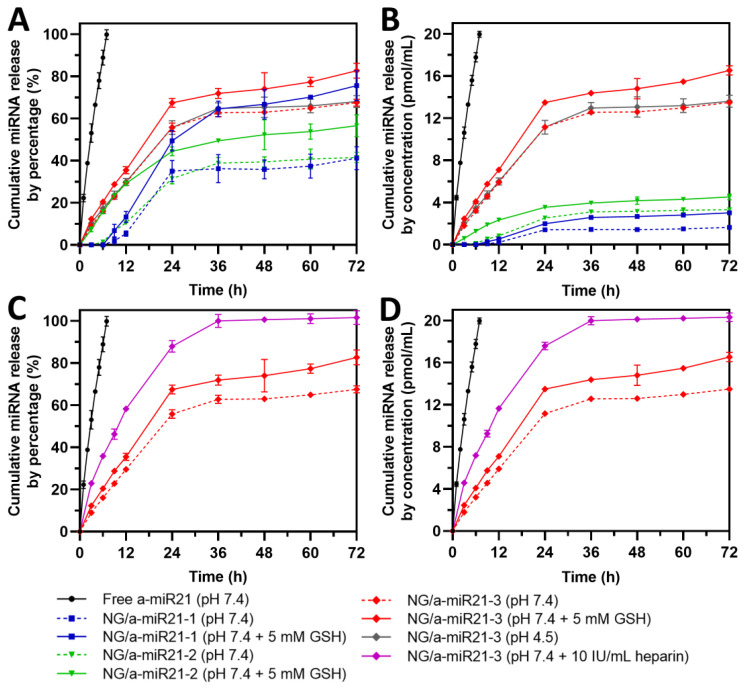
miRNA release profile by (**A**,**C**) percentage and (**B**,**D**) concentration from NG/a-miR21-1,2,3 at a nanogel concentration of 100 µg/mL with or without GSH treatment (5 mM GSH) in PBS (pH 7.4) at 37 °C for 72 h. NG/a-miR21-3 was also incubated in PBS (pH 4.5) and heparin-containing PBS (pH 7.4) at a concentration of 10 IU/mL. Free a-miR21 was equal to a-miR21 content in NG/a-miR21-3 to check the suitability of miRNA dialysis using a membrane with a molecular weight cutoff (MWCO) of ~100 kDa. Data are reported as the mean ± SD (n = 4).

As seen from Figure 5A, NG/a-miR21-1,2 had a delayed miRNA release in the normal condition (pH 7.4) until 9 h (~1.9% miRNA release). In contrast, NG/a-miR21-3 with a lower N/P ratio of 2 already showed a significant release at 3 h (~9.0% miRNA release), which was probably due to the weaker electrostatic interaction. On the other hand, in cancer-mimicking environments containing GSH, NG/a-miR21-1 showed enhanced release detected after 9 h (~6.8% miRNA release), and a significant release was observed from NG/a-miR21-2 in 3 h (~7.4% miRNA release) compared to that in pH 7.4 not containing GSH. At the same time, no significant change in the rate of miRNA release from NG/a-miR21-3 was observed in the medium with or without GSH. 

After 24 h, miRNA release from NG/a-miR21-1,2,3 reached 35.0%, 31.6%, and 55.8%, respectively, in normal physiological conditions, whereas in GSH environments, the release was enhanced to 49.4%, 44.4%, and 67.4%, respectively. The results show that the hydrolysis of disulfide bonds and disintegration of the nanogel network in the presence of GSH had only a minor effect on the weakening of the electrostatic interaction between the nanogel cation units and the miRNA phosphate groups. However, it can be seen that this effect was greatest for the sample with the highest N/P ratio (NG/a-miR21-1). In this case, the miRNA was released much faster when GSH was present (Figure 5A). For samples with a lower N/P ratio (NG/a-miR21-2 and NG/amiR21-3), only a small difference in miRNA release was observed after 72 h, whether GSH was present or not.

To be able to comprehensively compare the miRNA release behavior from NG/a-miR21-1,2,3, we also presented the release profile by concentration (pmol/mL) which can be seen in Figure 5B. It is visible that NG/a-miR21-3 had far more preferable miRNA release after 72 h (~16.5 pmol/mL) than NG/a-miR21-1 and NG/a-miR21-2 (~3.0 and ~4.5 pmol/mL, respectively). The release of miRNA was proportional to the miRNA content of the nanogels (MLC). 

No significant difference was observed in miRNA release between acidic (pH 4.5) and neutral conditions (pH 7.4), which was attributed to the lack of a pH-responsive component in the nanogel (Figure 5A,B, dotted red curve vs. solid gray curve).

Biologic stability of nanoplexes is a prerequisite for successful transfection. To show the reversibility of the electrostatic interactions, strongly negatively charged heparin was added to NG/miRNA dispersions, which resulted in a significantly accelerated release of miRNA. The addition of heparin in a concentration of 10 IU/mL to the release medium resulted in the dissociation of miRNA/nanogel complexes, also demonstrating the reversibility of the complexation. The complete release of miRNA from NG/a-miR21-3 was observed after 36 h in the presence of heparin. It is worth mentioning that, in an environment without the presence of heparin, the complete release of miRNA was not observed during the experiment.

In addition, miRNA release kinetics from NG/a-miR21-3 in pH 7.4 + 5 mM GSH were fitted to five different models. The results indicated that the release kinetics were prone to follow Higuchi and first-order models (R^2^ = 0.9460 and 0.9240, respectively) (Figure S11).

### 2.3. Cell Uptake

**Figure 6:**
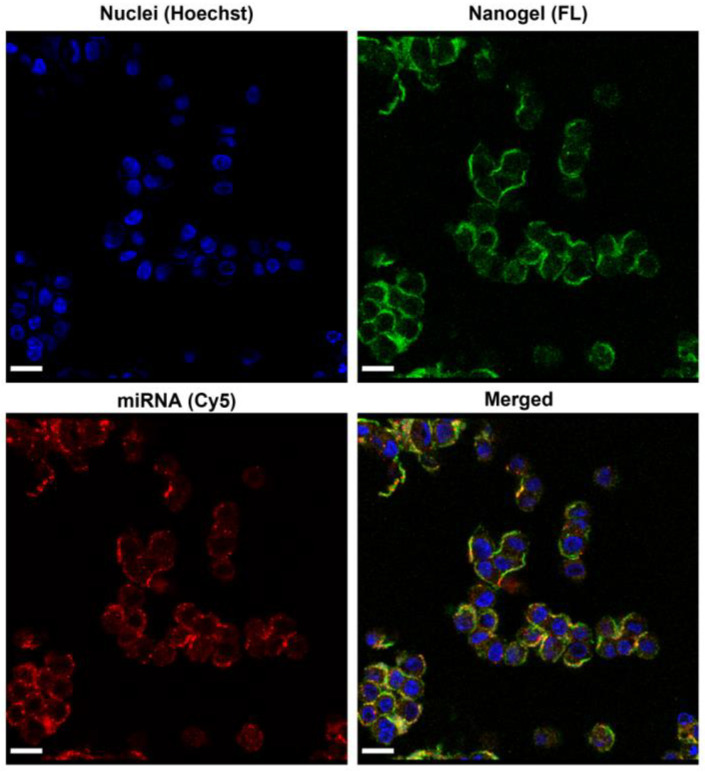
Cell uptake of FL-NG/Cy5-a-miR21-3 in HCT 116 colon cancer cell line (concentration: 500 µg/mL). Blue color indicates cell nuclei, green color indicates NG, and red color indicates miRNA. Scale bars = 20 μm.

To observe the uptake of NG/miRNA by the cells, NG labeled with fluorescein (FL, green fluorescence) and miRNA labeled with Cy5 (red fluorescence) were used. Figure 6 shows the confocal laser scanning microscope images of the HCT 116 colon cancer cells after 3 h incubation with FL-NG/Cy5-a-miR21-3. FL-NG/Cy5-a-miR21-3 was effectively taken up by the cancer cells, which proved its ability to deliver miRNA into cells.

### 2.4. Cytotoxicity Profile

**Figure 7:**
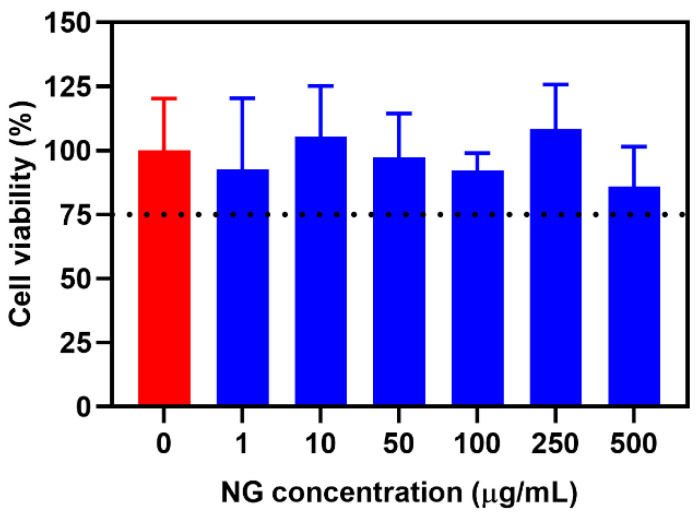
Cytotoxicity profile of bare NG in HCT 116 colon cancer cell line at different NG concentrations (0, 1, 10, 50, 100, 250, and 500 µg/mL) after 24 h of incubation at 37 °C. Data are presented as the mean ± SD (n = 4).

The cytotoxicity profile of bare NG was assessed in the HCT 116 colon cancer cell line, measured by cell viability (Figure 7). Bare NG had tolerable cytotoxicity (>75% cell viability) up to 500 µg/mL, indicating its suitability to be used as a safe miRNA delivery carrier.

## 3. Materials and Methods

### 3.1. Materials and Reagents

#### 3.1.1. Materials and Reagents for GSH-Responsive Nanogel Synthesis

The materials and reagents used were as follows: anti-miR21 oligonucleotides (a-miR21) and cyanine5 (Cy5)-tagged a-miR21 (sequences T*CAACCATCAGTCTGATAAGC*T*A and Cy5-T*CAACCATCAGTCTGATAAGC*T*A, Future Synthesis), heparin sodium salt (IU ≥ 100/mg, MW: 8–25 kDa, Alfa Aesar, Haverhill, MA, USA), (*N*,*N*-dimethylacrylamide (DMAM, Sigma Aldrich, St. Louis, MO, USA), [2-(acryloyloxy)ethyl]trimethylammonium chloride (ATC, Sigma Aldrich), *N*,*N*’-bis(acryloyl)cystamine (CBA, Alfa Aesar), lithium phenyl (2,4,6-trimethylbenzoyl)phosphinate (LAP, Carbosynth, Compton, UK), fluorescein *O*-acrylate (Sigma Aldrich), Span 80 (Sigma Aldrich), cyclohexane (Chempur, Karlsruhe, Germany), acetone (Chempur), dimethyl sulfoxide (DMSO, Fisher Bioreagents, Pittsburgh, PA, USA), and dialysis membrane (Spectrum™ Spectra/Por™ 2 RC Dialysis Membrane, MWCO: 100 kDa).

#### 3.1.2. Materials and Reagents for Cell Culture and In Vitro Assays

The HCT 116 colon cancer cell line (catalog no. CCL-247) was obtained from the American Type Culture Collection (ATCC, Manassas, VA, USA), Dulbecco’s modified Eagle medium (DMEM, PAN Biotech, Aidenbach, Germany), Roswell Park Memorial Institute 1640 (RPMI, PAN Biotech), fetal bovine serum (FBS, PAN Biotech), PBS (PAN Biotech), CCK-8 kit (Bimake, Houston, TX, USA), and Hoechst 33342 cell nuclei staining (Thermo Fisher Scientific, Waltham, MA, USA).

#### 3.1.3. General Methods

Ultrasonication at two different amplitudes: 40% and 60% using Sonics VCX 130 (Sonics & Materials, Inc., Newton, CT, USA) was conducted for redispersing nanogel powder and creating a water-in-oil (w/o) mini-emulsion, respectively. Purification of bare nanogels and NG/miRNA was achieved by a dialysis method following lyophilization in a freeze-dryer (ALPHA 1-2 LDplus, CHRIST) under 0.035 mbar at −50 °C. Deionized water (DI water) was produced using a reverse osmosis system (conductivity < 2 μS/cm).

### 3.2. Synthesis of GSH-Responsive Nanogels and Fluorescently Labeled Nanogels

Degradable nanogels were synthesized via free-radical polymerization (FRP) in an inverse w/o mini-emulsion according to our previous method [24,25]. A w/o mini-emulsion (10:1, *v*/*v*) was composed of 10.0 mL of cyclohexane containing Span-80 (600 mg) and 1.0 mL of PB solution (pH 7.0) containing monomers. The following steps were taken to synthesize nanogels: an aqueous phase was prepared in a 4 mL dark vial by adding CBA (26.0 mg, 0.10 mmol), with the main monomers DMAM and ATC (amounts specified in Table S1), and 0.2 M PB (pH 7.0) containing DMSO (10% *v*/*v*). The monomers were dissolved completely with a vortex. Then, the solution of the LAP initiator (2.3 mg, 0.008 mmol) was added. To create a mini-emulsion, the organic phase and aqueous phase were mixed and ultrasonicated at 60% amplitude for 5 min at 4 °C. The vial was protected from light with aluminum foil and placed over high-power light-emitting diodes (LEDs, 3 W, 395–405 nm) following photoirradiation for 30 min. To obtain nanogel powder, the suspension of nanogels was precipitated in 40 mL of cold acetone, centrifuged at 11,000 rpm for 10 min, and washed twice before air-drying overnight. On the next day, crude nanogels were dialyzed in a 100 kDa dialysis membrane against DI water. Dialysis was performed for 24 h with multiple media changes. Lastly, pure nanogel was frozen at −80 °C and lyophilized to obtain the nanogel powder. Fluorescently labeled nanogels (FL-NG) were synthesized with the same procedure but adding 0.5% *w*/*w* fluorescein *O*-acrylate in the aqueous phase.

### 3.3. miRNA Loading into Cationic Nanogels

The miRNA was loaded into cationic nanogels in a straightforward method. Briefly, bare nanogel powder (amounts specified in Table S2) was redispersed with 700 µL of water and sonicated at 40% amplitude for 30 s. Then, 100 µL of a-miR21 or Cy5-a-miR21 solution (concentration: 100 pmol/µL or equal to 0.725 µg/µL) was added to the nanogel dispersion. Three samples differing by N/P ratios of 10, 5, and 2 (mol/mol) were prepared to produce NG/a-miR21-1, NG/a-miR21-2, and NG/a-miR21-3, respectively. The mixtures containing relevant amounts of NG and a-miR21 were stirred overnight at 400 rpm under dark conditions. Then, dialysis was performed in a dialysis capsule (QuixSep^®^, 1 mL) using a dialysis membrane (MWCO 100 kDa) against 80 mL of DI water for 4 h. Finally, pure NG/a-miR21-1,2,3 was lyophilized and stored as a powder.

### 3.4. Nanogel Characterization

#### 3.4.1. Measurement of miRNA Loading Capacity (MLC) and miRNA Loading Efficiency (MLE)

The measurements of MLC and MLE were based on the fluorescence intensity, using a standard curve of Cy5-a-miR21 in PBS (R^2^ = 1.000, Ex: 650 nm, Em: 675 nm). Briefly, lyophilized pure nanogel powders of NG/a-miR21-1,2,3 were redispersed in water at a concentration of 1.0 mg/mL, following dilution with PBS (pH 7.4) to a concentration of 25 µg/mL. Then, the fluorescence intensity of dispersions was measured and converted to the mass of a-miR21. MLC and MLE were then calculated using the following equations:

$$\mathrm{MLC}\;(\%)=\frac{Actual\;loaded\;miRNA\;\left(mg\right)}{NG/miRNA\;\left(mg\right)}\times 100,$$

$$\mathrm{MLE}\;(\%)=\frac{Actual\;loaded\;miRNA\;\left(mg\right)}{Initial\;feed\;of\;miRNA\;\left(mg\right)\;}\times 100.$$

#### 3.4.2. Dynamic Light Scattering (DLS)

The Z-average hydrodynamic diameter (d_H_) and polydispersity index (PdI) of freshly prepared bare NG and NG/a-miR21-1,2,3 (1.0 mg/mL) in 1 mM KCl solution were determined using dynamic light scattering (DLS) (Malvern, Zetasizer Nano 90S), the system was equipped with a 4 mV He–Ne ion laser (λ = 633 nm) as the light source at a scattering angle of 90°. Similarly, the ζ potentials of bare NG and NG/a-miR21-1,2,3 were measured using electrophoretic light scattering (ELS) measurements (Malvern, Zetasizer Nano ZC) in 1 mM KCl solution.

#### 3.4.3. Cryogenic Transmission Electron Microscopy (Cryo-TEM)

NG/a-miR21-3 and GSH-treated NG (concentration: 500 µg/mL) were observed under cryo-TEM using a Tecnai F20 X TWIN microscope (FEI Company, Hillsboro, OR, USA).

#### 3.4.4. Stability Study of Nanogel in Biological Media

The stability of bare NG and NG/a-miR21-3 was measured at working concentrations (100 µg/mL) in different biological media, namely, water, PBS (pH 7.4), normal saline (0.90% *w*/*v* of NaCl), RPMI + 10% FBS, and DMEM + 10% FBS, at 37 °C for 5 days. The colloidal stability was observed visually, and the optical density (OD) was measured at 650 nm. The OD value was converted to percentage transmittance using the following equation:

Transmittance (%) = antilog (2 − absorbance).

#### 3.4.5. miRNA Release Study

The miRNA release from NG/a-miR21-1,2,3 was analyzed using the dialysis method. Initially, a dispersion of nanogel (concentration: 100 µg/mL) was prepared in water. Then, 800 µL of NG/a-miR21-1,2,3 was placed into the dialysis capsule (QuixSep^®^, 1 mL) using a dialysis membrane (MWCO 100 kDa). Free a-miR21 at the same miRNA concentration as NG/a-miR21-3 was also prepared to evaluate the release of free miRNA (~7 kDa) through the 100 kDa membrane. The dialysis capsules were immersed in 40 mL of PBS (pH 7.4 with 0 or 5 mM GSH) and incubated at 37 °C under continuous shaking (110 rpm). In addition, NG/a-miR21-3 was also incubated in PBS (pH 4.5) to analyze the release profile in acidic conditions. Samples of miRNA release (400 µL, dissolution medium) were taken at predetermined time intervals (3, 6, 9, 12, 24, 36, 48, 60, and 72 h) and replaced with the same amount of prewarmed fresh medium. The amount of released miRNA in the withdrawn samples was determined by fluorescence analysis (Ex/Em max of 650/675 nm) converted to miRNA concentration using a standard curve (R^2^ = 1.000).

The miRNA release from NG/a-miR21-3 was also evaluated in the presence of a competing polyanion, heparin, at a concentration of 10 IU/mL.

### 3.5. Cell Culture and In Vitro Study

#### 3.5.1. Cell Uptake Study by Confocal Microscopy

HCT 116 cancer cell lines (5 × 10^3^ cells/well) were seeded in µ-Slide eight-well glass-bottom plates (ibidi, USA) in 200 µL of DMEM supplemented with 10% FBS and incubated at 37 °C for 24 h. Then, the medium was replaced with fresh DMEM + 10% FBS containing FL-NG/Cy5-a-miR21-3 (500 μg/mL) and cocultured for 3 h. FL (green) represents NG and Cy5 (red) represents miRNA. Cell nuclei were then stained with Hoechst 33342, 30 min before observation by confocal laser scanning microscopy (CLSM, Olympus FluoView FV1000, ZEISS, Dublin, CA, USA).

#### 3.5.2. Cytotoxicity Study

The cytotoxicity profile of bare NG was assessed in HCT 116 cancer cell line using a standard CCK-8 assay. HCT 116 cells (5 × 10^3^ cells/well) were seeded in 96-well plates in 100 μL of DMEM supplemented with 10% FBS and 1% antibiotics for 24 h, following incubation with bare nanogels at different concentrations (0, 1, 10, 50, 100, 250, and 500 μg/mL). CCK assay was performed by adding 10 μL of CCK-8 solution in each well following incubation at 37 °C for 2 h. The absorbance was then measured by a microplate reader at 450 nm. The relative cell viability (%) was expressed as a fraction of the percentage of cell growth occurring in the presence of nanogel vs. the absence of nanogel (control).

### 3.6. Statistical Analysis

GraphPad Prism Version 6.0 software (GraphPad, San Diego, CA, USA) was used for the statistical analysis. Data analysis was performed using one-way analysis of variance (ANOVA). The significance level was set at *p* < 0.05, with all data displayed as mean ± SD (*n* = 4).

## 4. Conclusions

Synthesis of acrylamide-based nanogels, containing a disulfide crosslinker via photoinitiated FRP in an inverse microemulsion technique, was developed. The successful encapsulation of miRNA into nanogels was carried out by self-assembly of nanogel cationic groups and miRNA anionic phosphate groups by electrostatic complexation. The miRNA-loaded nanogels characterized by a spherical shape (d_H_ about 100 nm) and a moderate positive charge (ζ potential in the range of 12–24 mV) were able to form stable dispersions in various biological media, including serum-enriched media. The miRNA release studies demonstrated that the presence of GSH in the release environment had only a minor effect on miRNA release. Instead, a markedly accelerated release of miRNA was observed in the presence of a strong heparin polyanion, indicating the reversibility of the complexation. The miRNA-loaded nanogels were efficiently absorbed by HCT 116 cancer cells within a short incubation time and showed tolerable cytotoxicity, making them a promising miRNA delivery system.

## Figures and Tables

**Table 1:** Physicochemical properties of bare nanogels and after loading with a-miR21 at different N/P ratios.

Nanogels	N/P Ratio	MLC (%)	MLE (%)	d_H_ (PdI) (nm)	ζ Potential (mV)
NG	-	-	-	92.5 (0.43)	27.4
NG/a-miR21-1	10	1.4	100.0	109.2 (0.34)	24.2
NG/a-miR21-2	5	2.8	100.0	116.8 (0.36)	20.8
NG/a-miR21-3	2	6.7	99.7	122.1 (0.46)	11.9

## Data Availability

Not applicable.

## Supplementary Materials

The following supporting information can be downloaded at https://www.mdpi.com/article/10.3390/molecules28020761/s1: Table S1. Monomer feed composition based on moles and mass; Table S2. Post-polymerization loading; Figure S1. Standard curve of Cy5-a-miR21 in PBS (pH 7.4); Figure S2. Fluorescence spectra of Cy5-a-miR21 and NG/Cy5-a-miR21 in PBS (pH 7.4); Figures S3–S6. Average size distributions (d_H_) of bare NG, NG/a-miR21-1, NG/a-miR21-2, and NG/a-miR21-3, respectively; Figures S7–S10. Average ζ potentials of bare NG, NG/a-miR21-1, NG/a-miR21-2, and NG/a-miR21-3, respectively; Figure S11. NG/a-miR21-3 release data in pH 7.4 + 5 mM GSH fitted to zero-order, first-order, Korsmeyer–Peppas, Higuchi, and Hixon–Crowell models, respectively.
