# Supplementary material for: A Simple Synthesis of Reduction-Responsive Acrylamide-Type Nanogels for miRNA Delivery
Source: Molecules. 2023 Jan 12;28(2):761. doi: 10.3390/molecules28020761 (PMC9861385; doi:10.3390/molecules28020761)
Supplement: Supplementary file 1 [file molecules-28-00761-s001.zip › molecules-2080213-supplementary.pdf]

Supplementary Information

for

A Simple Synthesis of Reduction-Responsive Acrylamide-Type Nanogels for miRNA Delivery

Ali Maruf [1,2], Małgorzata Milewska [1,2], Anna Lalik [2,3], Sebastian Student [2,3] and Ilona Wandzik [1,2,*]

[1] Department of Organic Chemistry, Bioorganic Chemistry and Biotechnology, Faculty of Chemistry, Silesian University of Technology, Krzywoustego 4, 44-100 Gliwice, Poland
[2] Biotechnology Center, Silesian University of Technology, Krzywoustego 8, 44-100 Gliwice, Poland
[3] Department of Systems Biology and Engineering, Faculty of Automatic Control, Electronics and Computer Science, Silesian University of Technology, Akademicka 16, 44-100 Gliwice, Poland
* Correspondence: ilona.wandzik@polsl.pl

Table S1. Monomer feed composition based on moles and mass

Formulation[a]	DMAM		ATC		CBA		LAP	
	mg	mmol	mg	mmol	mg	mmol	mg	mmol
NG	183.6	1.852	21.5	0.111	26.0	0.099	2.3	0.008

[a] Total volume of aqueos phase and organic phase were 1 mL and 10 mL, respectively.

Table S2. Post-polymerization loading

Nanogels	NG (mg)[a]	ATC (mmol)	N/P ratio
NG	-	-	-
NG/a-miR21-1	5.0	2.38	10
NG/a-miR21-2	2.5	1.19	5
NG/a-miR21-3	1.0	0.48	2

[a] miRNA was loaded at concentration of 100 pmol/μL (100 μL).

Standard curve of Cy5-a-miR21 in PBS (pH 7.4)

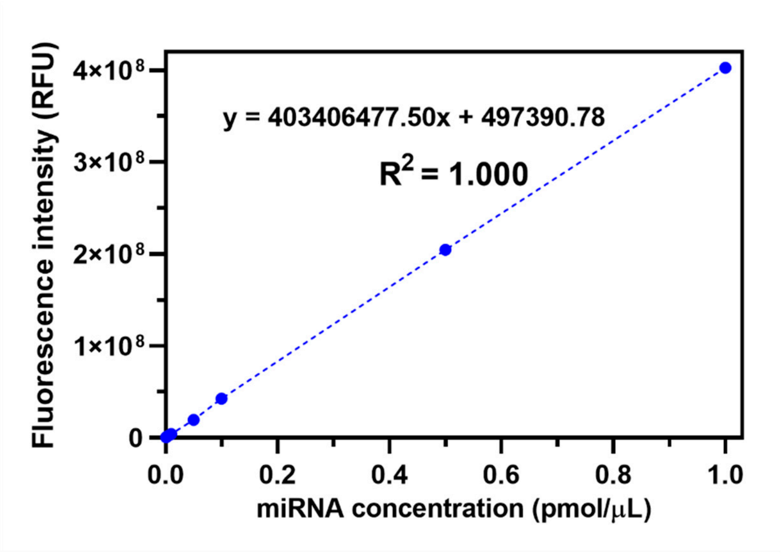

Figure S1. Standard curve of Cy5-a-miR21 in PBS (pH 7.4). The standard curve was created from diferent concentrations of a-miR21: 0.0005, 0.001, 0.005, 0.01, 0.05, 0.1, 0.5, and 1.0 pmol/μL. Data were reported as mean ± SD (n = 4).

Fluorescence spectra of Cy5-a-miR21 and NG/Cy5-a-miR21

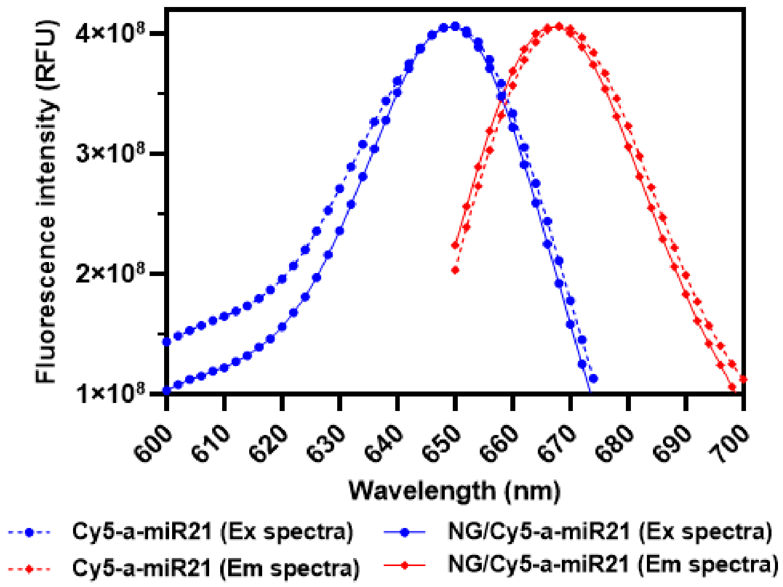

Figure S2. Fluorescence spectra of Cy5-a-miR21 and NG/Cy5-a-miR21 in PBS (pH 7.4). Both Cy5-a-miR21 and NG/Cy5-a-miR21 indicated the same Ex/Em max (regardless of a relatively different spectra particularly at 600–635 nm exitation area). For analysis purposes (standard curve generation, miRNA loading analysis, and miRNA release study) we used the maximum wavelengths: 650 and 675 nm for the Ex and Em, respectively.

DLS measurements of nanogels

			Size (d.nm):	% Intensity:	St Dev (d.nm):
Z-Average (d.nm):	92.47	Peak 1:	116.2	95.4	63.16
PdI:	0.429	Peak 2:	4985	3.4	667.3
Intercept:	0.974	Peak 3:	591.0	1.3	142.6
Result quality :	Good				

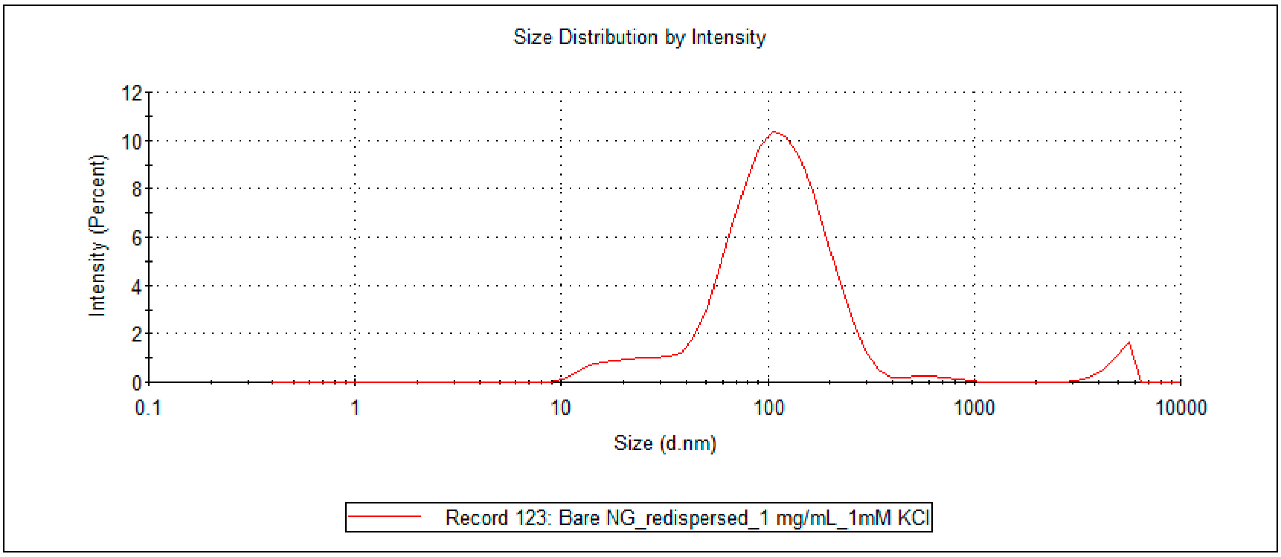

Figure S3. Average size distribution (d_H) of bare NG in 1 mM KCl from four replications.

			Size (d.nm):	% Intensity:	St Dev (d.nm):
Z-Average (d.nm):	109.2	Peak 1:	136.6	96.9	73.50
PdI:	0.341	Peak 2:	4792	3.1	840.9
Intercept:	0.965	Peak 3:	0.000	0.0	0.000
Result quality :	Good				

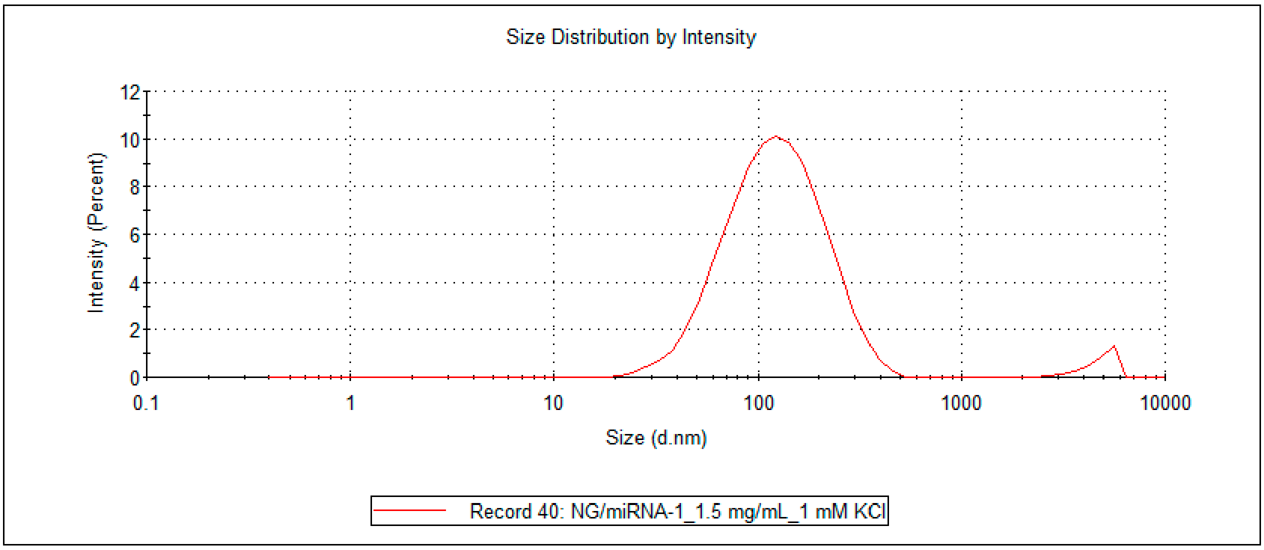

Figure S4. Average size distribution (d_H) of NG/a-miR21-1 in 1 mM KCl from four replications.

			Size (d.nm):	% Intensity:	St Dev (d.nm):
Z-Average (d.nm):	116.8	Peak 1:	144.5	96.5	77.32
PdI:	0.361	Peak 2:	4961	3.5	693.3
Intercept:	0.964	Peak 3:	0.000	0.0	0.000
Result quality :	Good				

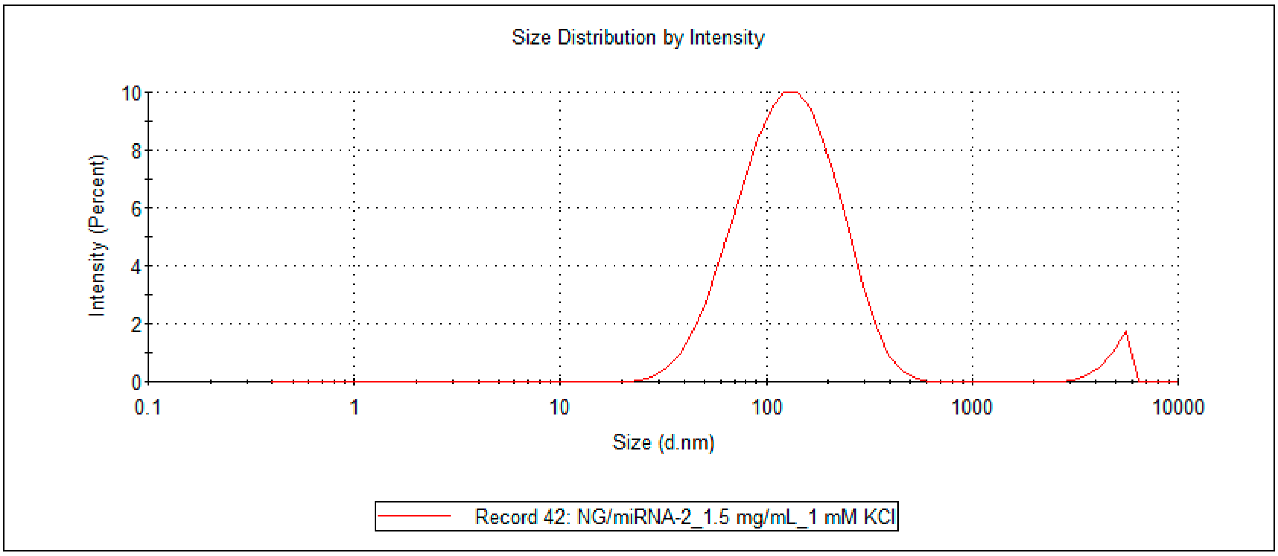

Figure S5. Average size distribution (d_H) of NG/a-miR21-2 in 1 mM KCl from four replications.

			Size (d.nm):	% Intensity:	St Dev (d.nm):
Z-Average (d.nm):	122.1	Peak 1:	191.3	95.1	160.9
PdI:	0.457	Peak 2:	4390	4.9	997.9
Intercept:	0.952	Peak 3:	0.000	0.0	0.000
Result quality :	Good				

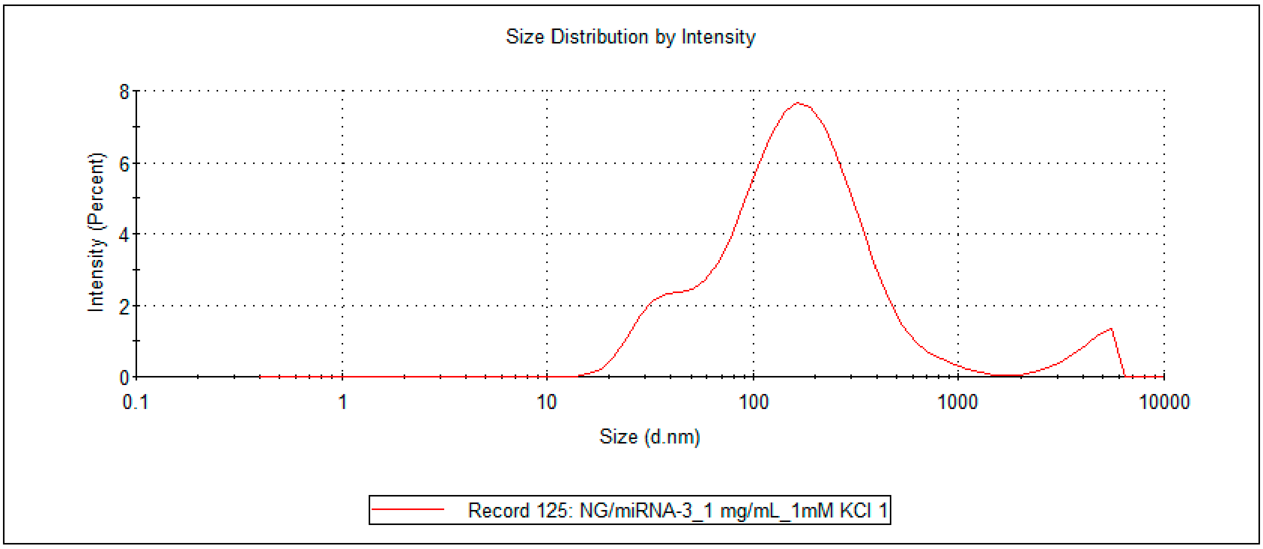

Figure S6. Average size distribution (d_H) of NG/a-miR21-3 in 1 mM KCl from four replications.

Zeta potential (ζ) measurements of nanogels

			Mean (mV)	Area (%)	St Dev (mV)
Zeta Potential (mV):	27.4	Peak 1:	27.4	100.0	3.69
Zeta Deviation (mV):	3.40	Peak 2:	0.00	0.0	0.00
Conductivity (mS/cm):	0.183	Peak 3:	0.00	0.0	0.00

Result quality : Good

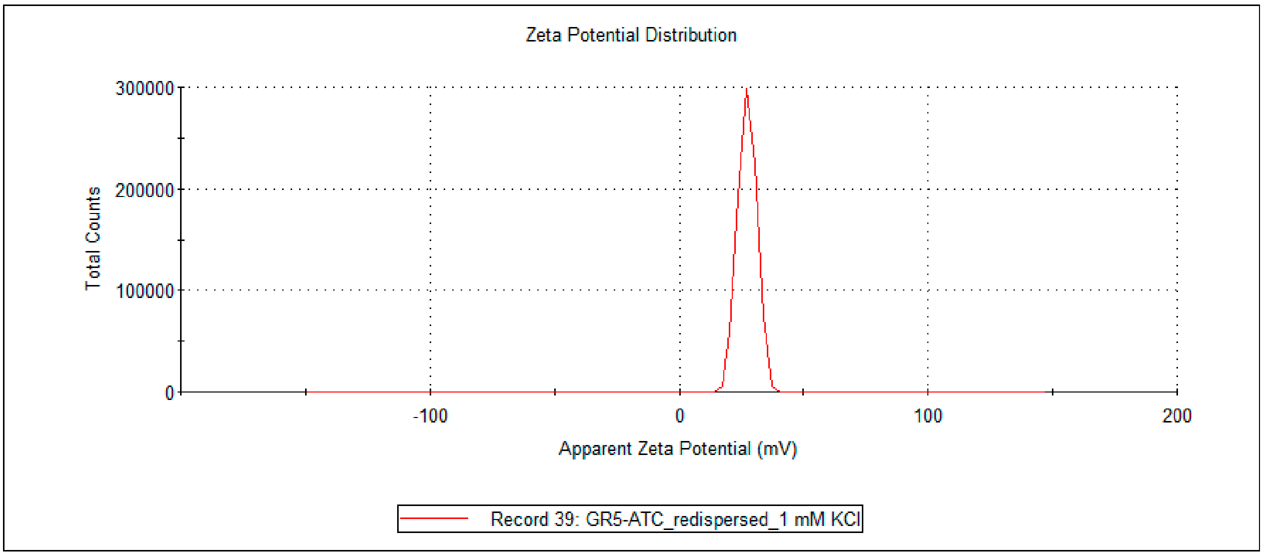

Figure S7. Average ζ potential of bare NG in 1 mM KCl from four replications.

			Mean (mV)	Area (%)	St Dev (mV)
Zeta Potential (mV):	24.2	Peak 1:	24.2	100.0	3.54
Zeta Deviation (mV):	3.43	Peak 2:	0.00	0.0	0.00
Conductivity (mS/cm):	0.187	Peak 3:	0.00	0.0	0.00

Result quality : Good

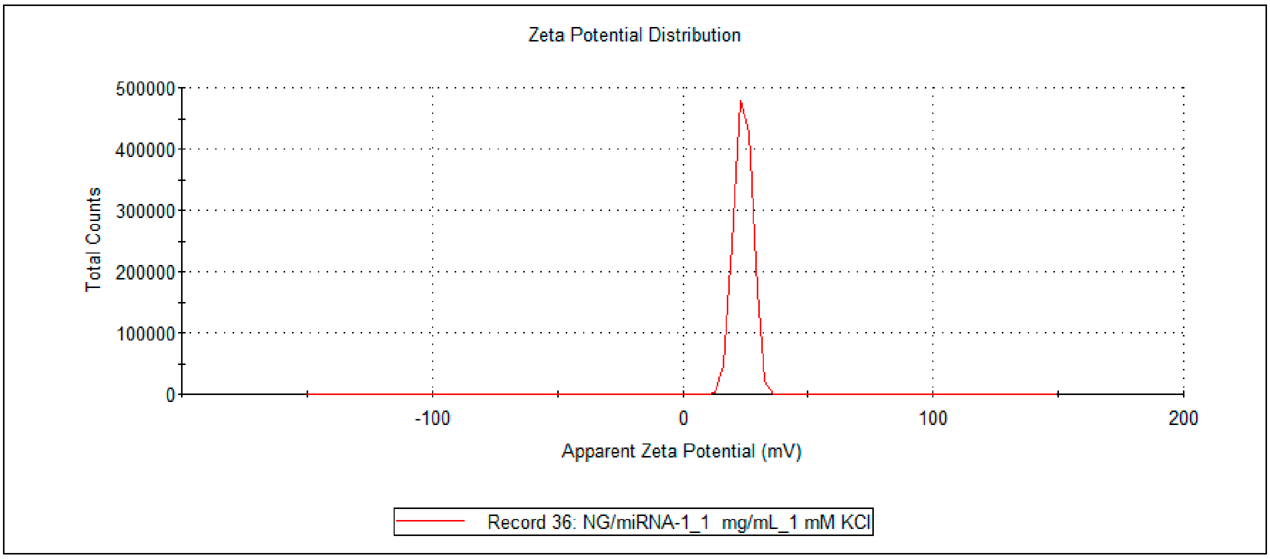

Figure S8. Average ζ potential of NG/a-miR21-1 in 1 mM KCl from four replications.

			Mean (mV)	Area (%)	St Dev (mV)
Zeta Potential (mV):	20.8	Peak 1:	20.8	100.0	4.93
Zeta Deviation (mV):	4.71	Peak 2:	0.00	0.0	0.00
Conductivity (mS/cm):	0.221	Peak 3:	0.00	0.0	0.00

Result quality : Good

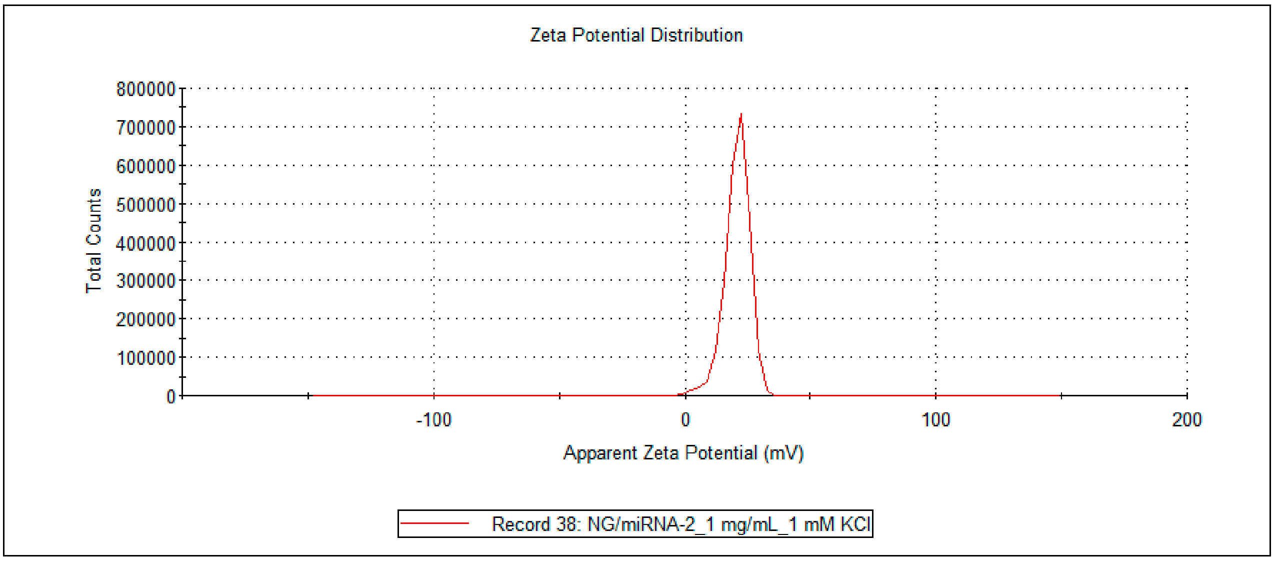

Figure S9. Average ζ potential of NG/a-miR21-2 in 1 mM KCl from four replications.

			Mean (mV)	Area (%)	St Dev (mV)
Zeta Potential (mV):	11.9	Peak 1:	12.0	100.0	6.07
Zeta Deviation (mV):	6.04	Peak 2:	0.00	0.0	0.00
Conductivity (mS/cm):	0.170	Peak 3:	0.00	0.0	0.00

Result quality : Good

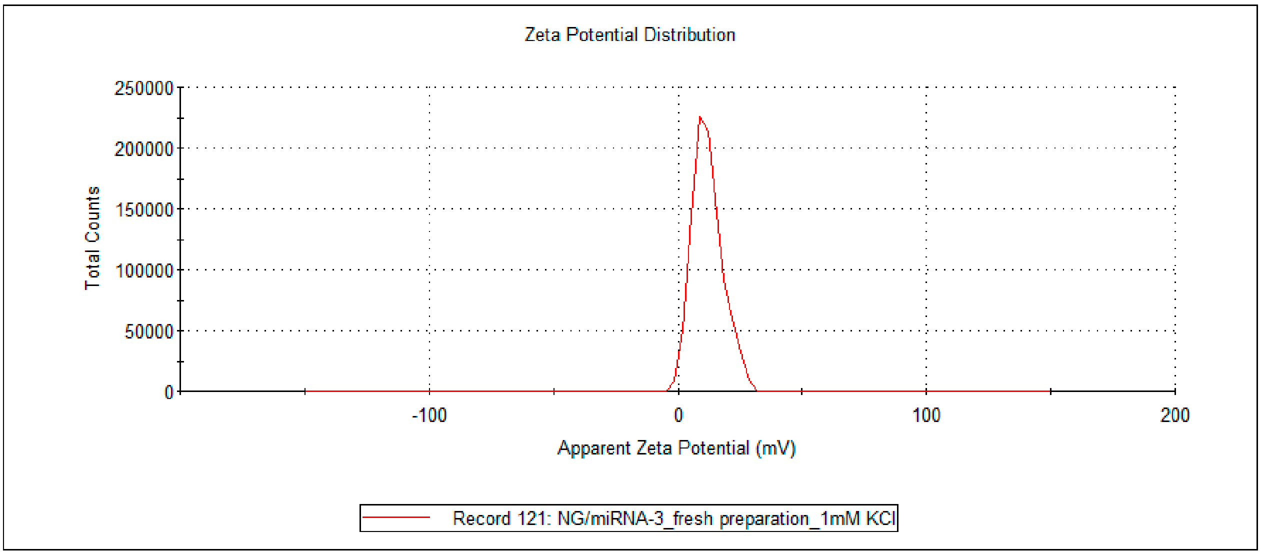

Figure S10. Average ζ potential of NG/a-miR21-3 in 1 mM KCl from four replications.

MiRNA release kinetics from nanogels

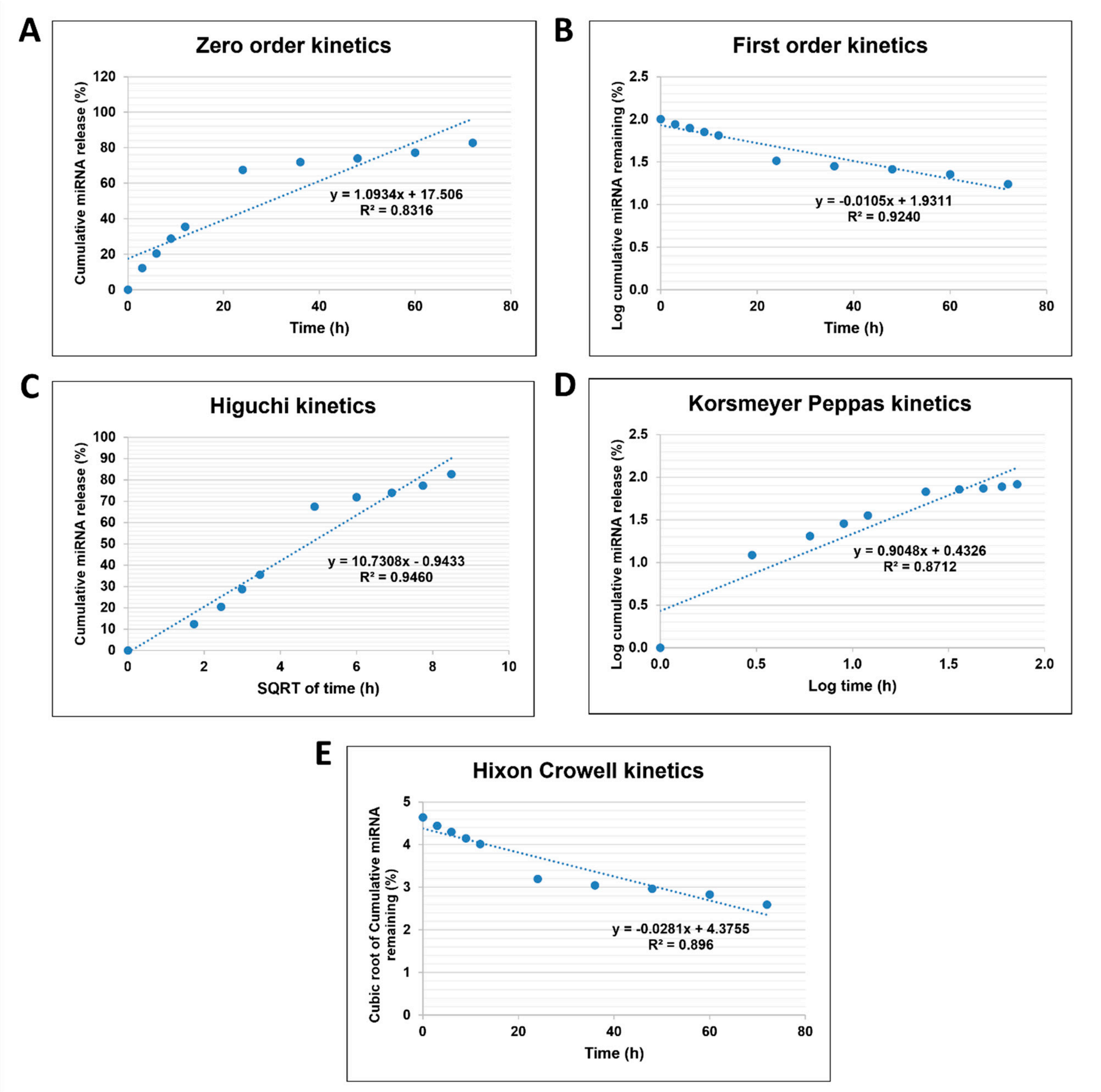

Figure S11. NG/a-miR21-3 release data in pH 7.4 + 5 mM GSH fitted to (A) zero order, (B) first order, (C) Higuchi, (D) Korsmeyer Peppas, and (E) Hixon Crowell models.
